# Patterns of Mobile Phone Ownership and Use Among Pregnant Women in Southern Tanzania: Cross-Sectional Survey

**DOI:** 10.2196/17122

**Published:** 2020-04-08

**Authors:** Lavanya Vasudevan, Jan Ostermann, Sara Marwerwe Moses, Esther Ngadaya, Sayoki Godfrey Mfinanga

**Affiliations:** 1 Department of Family Medicine and Community Health School of Medicine Duke University Durham, NC United States; 2 Duke Global Health Institute Duke University Durham, NC United States; 3 Center for Health Policy and Inequalities Research Duke University Durham, NC United States; 4 Department of Health Services, Policy & Management Arnold School of Public Health University of South Carolina Columbia, SC United States; 5 South Carolina SmartState Center for Healthcare Quality University of South Carolina Columbia, SC United States; 6 Muhimbili Research Centre National Institute for Medical Research Dar-es-Salaam United Republic of Tanzania; 7 Muhimbili University of Health and Allied Sciences Dar-es-Salaam United Republic of Tanzania; 8 School of Life Sciences and Bioengineering Nelson Mandela African Institution of Science and Technology Arusha United Republic of Tanzania

**Keywords:** digital health, mobile health, pregnant women, Tanzania

## Abstract

**Background:**

There is a paucity of subnational data on patterns of mobile phone ownership and use in Tanzania to inform the development of digital health interventions.

**Objective:**

The aim of this study is to assess patterns of mobile phone ownership and use in pregnant women to inform the feasibility and design of digital health interventions for promoting timely uptake of childhood vaccines in southern Tanzania.

**Methods:**

Between August and November 2017, pregnant women in their third trimester were enrolled at health facilities and from surrounding communities, and asked about their patterns of mobile phone ownership and use in an interviewer administered survey.

**Results:**

Of 406 women, only 3 had never used a phone. Most women (>98%) could make and receive phone calls. Compared to urban women, rural women reported higher mobile phone use rates but were less likely to be sole owners of phones, and less likely to send or receive SMS, transact money, browse the internet, or use social media via mobile phones.

**Conclusions:**

The findings suggest high feasibility for digital health interventions delivered via mobile phones to pregnant women in southern Tanzania. The feasibility of smartphone-based interventions or strategies relying on the use of social media or the internet is limited.

## Introduction

In recent years, many efforts have leveraged increasing mobile-cellular subscription rates in low- and middle-income countries (LMIC) as a mechanism to promote childhood vaccinations [1,2]. Examples of “digital health” strategies for promoting childhood vaccinations in LMICs include text message–based delivery of educational content, appointment reminders, conditional financial transfers, and tools to support health care providers in vaccination delivery [1,2]. The feasibility, successful implementation, and scale-up of these digital health strategies depends on the availability of mobile-cellular infrastructure and patterns of mobile phone ownership and use in target populations. Mobile-cellular infrastructure and patterns of mobile use also provide insights about the feasibility and potential success of next-generation interventions and technologies (eg, smartphone apps, wearables).

The government of Tanzania has developed a detailed investment road map for the use of digital health interventions to strengthen the performance of the national health system [3]. Despite ranking 123rd among 143 countries in its Network Readiness Index, a measure summarizing the extent that countries benefit from the opportunities provided by information and communication technologies, Tanzania currently has the third highest mobile cellular subscription rate in East Africa at 77 per 100 people [4,5]. In addition, several large text messaging programs have been successfully implemented to deliver health information to target populations via mobile phones. Examples of such text messaging programs include the *Wazazi Nipendeni* multimedia campaign and the *mobile for reproductive health* educational messages for reproductive health [6,7] Yet, there is little subnational data on mobile phone ownership and use that are publicly available to inform the design and feasibility of digital health interventions.

In support of diverse research and intervention studies in urban and rural areas in Tanzania, we sought to develop a *mobile phone-assisted reminder and incentive system (mPARIS),* a digital health system capable of, among other things, sending reminders and conditional financial transfers to mothers of newborn children as a means of promoting timely uptake of childhood vaccines [8,9]. To inform the feasibility and design of digital health interventions using *mPARIS*, we used a structured survey to assess pregnant women’s mobile phone ownership and use in the Mtwara region in southern Tanzania. The findings of the survey are reported below.

## Methods

Mobile phone ownership and use were evaluated to inform research on the feasibility and potential efficacy of SMS reminders and conditional financial incentives for improving the timeliness of childhood vaccinations in southern Tanzania. The study protocol for the parent study, including objectives, context, and methods, was previously published [8]. The protocol was registered in ClinicalTrials.gov (Protocol NCT03252288) and approved by the Institutional Review Boards at Duke University (Protocol 2017-0591) and the University of South Carolina (facilitated review, Pro00051213) in the United States, and the National Institute for Medical Research (NIMR) in Tanzania (NIMR/HQ/R.8a/Vol. IX/2194). Methods pertinent to the analysis of mobile phone ownership and use are presented below.

The study was implemented in one rural and one urban district in the Mtwara region in southern Tanzania. In 2015, Mtwara ranked 13th in the Human Development Index among Tanzania’s 21 regions. Between August and November 2017, pregnant women were recruited from 4 urban and 8 rural health facilities and their surrounding communities. Eligibility was limited to health facilities that regularly provide childhood vaccinations.

To participate in the study, women had to meet the following inclusion criteria: be 16 years or older, be in the third trimester of pregnancy, have access to a mobile phone, and provide informed consent. Eligible women receiving antenatal care at participating facilities were approached by trained study personnel and offered enrollment in the study. Participating women and local community leaders were asked to identify other pregnant women in their community, who, if eligible, were also offered enrollment in the study. Informed consent was obtained from all participating women. Upon enrollment, a trained research assistant administered a structured survey comprised of closed-ended questions to collect data on sociodemographic characteristics, reproductive and antenatal care history, and mobile phone ownership and use. Data were collected electronically using the Qualtrics (Provo, UT) survey platform installed on tablet devices.

Data were imported into the Stata version 16 (StataCorp, College Station, TX) statistical software for analysis. Descriptive summaries (means, standard deviations, and percentages) were calculated for relevant survey responses and are presented below. Differences between rural and urban women were evaluated using the 2-tailed Student’s *t* test for continuous variables and chi-square tests for categorical variables.

## Results

A total of 406 pregnant women were enrolled in the study. Table 1 summarizes their demographic characteristics, and Table 2 details their mobile phone ownership and use. Enrolled women were 28 years of age, on average. A majority of the women were married, had at least standard 7 schooling, and were either employed or self-employed. Education and employment distributions differed between rural and urban women. A majority of women had started their antenatal care in the second trimester and most had previously given birth.

**Table 1:** Characteristics of pregnant women in Southern Tanzania, 2017.

Characteristics		All (N=406)	Urban (n=212)	Rural (n=194)	*P* value^a^
Age (years), mean (SD)		27.9 (7.2)	27.5 (6.5)	28.4 (7.9)	.19
**Marital status, n (%)**					.42
	Married	331 (81.5)	174 (82.1)	157 (80.9)	
	Widowed	2 (0.5)	1 (0.5)	1 (0.5)	
	Divorced/separated	20 (4.9)	7 (3.3)	13 (6.7)	
	Never married	53 (13.1)	30 (14.2)	23 (11.9)	
**Employment, n (%)**					<.001
	Unemployed/housewife	165 (40.6)	93 (43.9)	72 (37.1)	
	Self-employed	209 (51.5)	94 (44.3)	115 (59.3)	
	Employed	25 (6.2)	22 (10.4)	3 (1.5)	
	Other (eg, student, casual laborer)	7 (1.7)	3 (1.4)	4 (2.1)	
**Education, n (%)**					<.001
	None	103 (25.4)	24 (11.3)	79 (40.7)	
	Standard 1-6	31 (7.6)	13 (6.1)	18 (9.3)	
	Standard 7	205 (50.5)	123 (58.0)	82 (42.3)	
	Form 1-4	54 (13.3)	40 (18.9)	14 (7.2)	
	Form 5 or higher	13 (3.2)	12 (5.7)	1 (0.5)	
**First antenatal care visit, n (%)**					.86
	First trimester	159 (39.2)	83 (39.2)	76 (39.2)	
	Second trimester	219 (53.9)	114 (53.8)	105 (54.1)	
	Third trimester	11 (2.7)	7 (3.3)	4 (2.1)	
	Don't know	17 (4.2)	8 (3.8)	9 (4.6)	
**Reproductive history, n (%)**					.77
	First birth	91 (23.6)	48 (24.2)	43 (23.0)	
	Prior births	294 (76.4)	150 (75.8)	144 (77.0)	

^a^Denotes the statistical significance of differences between rural and urban participants.

**Table 2:** Mobile phone ownership and use among pregnant women in Southern Tanzania, 2017.

Survey Questions		All (N=406), n (%)	Urban (n=212), n (%)	Rural (n=194), n (%)		*P* value^a^
**How often do you use a mobile phone?**					<.001	
	Never	3 (0.7)	0 (0.0)	3 (1.5)		
	Less than once a week	325 (80.0)	195 (92.0)	130 (67.0)		
	At least once a week	52 (12.8)	12 (5.7)	40 (20.6)		
	Everyday	26 (6.4)	5 (2.4)	21 (10.8)		
**Who owns the mobile phone that you use?^b^**					<.001	
	Woman only	232 (57.6)	148 (69.8)	84 (44.0)		
	Father of the child to be born only	85 (21.1)	31 (14.6)	54 (28.3)		
	Woman and father of the child, jointly	27 (6.7)	13 (6.1)	14 (7.3)		
	Woman and other relative	8 (2.0)	6 (2.8)	2 (1.0)		
	Others	51 (12.7)	14 (6.6)	37 (19.4)		
**Have you ever used a mobile phone to:^b^**						
	Make phone calls	398 (98.8)	210 (99.1)	188 (98.4)		.57
	Receive phone calls	400 (99.3)	210 (99.1)	190 (99.5)		.62
	Send or receive SMS	311 (77.2)	182 (85.8)	129 (67.5)		<.001
	Receive money	300 (74.4)	172 (81.1)	128 (67.0)		.001
	Send money	248 (61.5)	154 (72.6)	94 (49.2)		<.001
	Browse the internet	32 (7.9)	30 (14.2)	2 (1.0)		<.001
	Use Facebook	37 (9.2)	31 (14.6)	6 (3.1)		<.001
	Use WhatsApp	34 (8.4)	31 (14.6)	3 (1.6)		<.001
	None of the above	1 (0.2)	1 (0.5)	0 (0.0)		.34
**If an important message were to be delivered to you on a mobile phone, how soon would you receive it?^b^**					.10	
	Same day	390 (96.8)	208 (98.1)	182 (95.3)		
	Next day	12 (3.0)	3 (1.4)	9 (4.7)		
	2-3 days	0 (0.0)	0 (0.0)	0 (0.0)		
	4-7 days	0 (0.0)	0 (0.0)	0 (0.0)		
	>7 days	1 (0.2)	1 (0.5)	0 (0.0)		
**In the past month, how often have you had problems with charging phones?^b^**					.17	
	Less than once per week	312 (77.4)	172 (81.1)	140 (73.3)		
	One or more times per week	82 (20.3)	36 (17.0)	46 (24.1)		
	Most days	9 (2.2)	4 (1.9)	5 (2.6)		
**In the past month, how often have you had connection problems?^b^**					.25	
	Less than once per week	361 (89.6)	193 (91.0)	168 (88.0)		
	One or more times per week	40 (9.9)	19 (9.0)	21 (11.0)		
	Most days	2 (0.5)	0 (0.0)	2 (1.0)		
**How much do you spend per week on phone charges?^b^**					<.001	
	0-499 TSH^c^	41 (10.2)	20 (9.4)	21 (11.0)		
	500-999 TSH	185 (45.9)	78 (36.8)	107 (56.0)		
	1000-1999 TSH	145 (36.0)	91 (42.9)	54 (28.3)		
	2000-4999 TSH	28 (6.9)	20 (9.4)	8 (4.2)		
	5000-9999 TSH	4 (1.0)	3 (1.4)	1 (0.5)		
**Do you use any bundles?^b^**						
	Daily	261 (64.8)	136 (64.2)	125 (65.4)		.79
	Weekly	169 (41.9)	106 (50.0)	63 (33.0)		<.001
	Monthly	6 (1.5)	4 (1.9)	2 (1.0)		.49
	None	50 (12.4)	16 (7.5)	34 (17.8)		.002

^a^Denotes the statistical significance of differences between rural and urban participants.

^b^Question skipped for 3 women who said they had never used a phone.

^c^TSH: Tanzanian shillings.

Only 3 women, all residing in rural areas, had never used a mobile phone. However, rural women reported more frequent mobile phone use than urban women. Most women either owned a mobile phone or could access one through family members including the father of the child to be born. Notably, fewer rural women reported being sole owners of phones compared to urban women. Similar percentages of urban and rural women reported using phones for making phone calls. However, compared to urban women, fewer rural women reported being able to communicate via text messaging and transact mobile money. Fewer than 10% of both urban and rural women reported using social media or the internet on phones.

Most urban and rural women said that important messages delivered to a mobile phone would reach them the same day. Problems related to phone charging and network connectivity were reported to be infrequent (less than once per week) by a majority of the women. Most women reported spending 500-2000 Tanzanian shillings (TSH; US $1 corresponded to approximately 2200 TSH at the time of the study) per week on phone-related charges.

## Discussion

Study findings suggest high mobile phone access among pregnant women in the Mtwara region. Our data suggests the feasibility of text- or voice-based interventions delivered via mobile phones to pregnant women in the Mtwara region of southern Tanzania, but there was less readiness for smartphone-based interventions or strategies relying on the use of social media or the internet. High rates of mobile phone use for financial transactions (sending or receiving money) and the widespread use of bundles, both of which require menu-based interactions with phones, suggest that Unstructured Supplementary Service Data (USSD) assessments or interventions may be feasible. Potential applications could include short questionnaires for data collection or monitoring. Due to observed variations in phone access and ownership, it is likely that any mobile phone–based interventions will be delivered to shared phones for some participants. This finding may be of relevance to studies involving sensitive health topics (eg, HIV/AIDS and other infectious diseases; intimate partner violence).

According to our data, the population in the Mtwara region are relatively smartphone-naive, compared to some other Tanzanian cities like Dar-es-Salaam and Arusha, where 4G network infrastructure has been previously reported [10]. Smartphone-based interventions may be possible in Mtwara, but may require larger financial investments for the provision of smartphones to participants, as well as higher training needs to facilitate their use. We did not formally assess whether mobile networks in the Mtwara region would be able to support broad use of smartphones.

This study is subject to several limitations. First, only women who had access to a mobile phone were enrolled for the survey. Our study, therefore, excludes women from families that do not have access to a mobile phone. Literature suggests that women from socioeconomically disadvantaged families are more likely to lack mobile phone access [10]. Such women may be more vulnerable and could potentially benefit more from interventions promoting health information and health service use. Second, the selected facilities were within 20 kilometers of Mtwara, the urban regional capital and commercial center. Generalizability of the study findings is limited to the specific study area in late 2017, as mobile phone use and ownership characteristics may vary in other regions and is likely to increase over time. Third, the study provides only contextual information for the design of interventions; qualitative work concerning end user perceptions on feasibility and acceptability should complement the design and implementation of specific digital health interventions.

In conclusion, study findings suggest high feasibility of text-, voice-, or USSD-based interventions delivered via mobile phones to pregnant women in the Mtwara region of southern Tanzania. The use of social media and internet among pregnant women is limited. Future studies may use this study’s findings to track growth in mobile phone ownership and changes in use patterns among pregnant women in the region.

## Abbreviations

LMIC: low- and middle-income countries
mPARIS: mobile phone-assisted reminder and incentive system
TSH: Tanzanian shilling
USSD: Unstructured Supplementary Service Data.
